# Structure-factor amplitude reconstruction from serial femtosecond crystallography of two-dimensional membrane-protein crystals

**DOI:** 10.1107/S2052252518014641

**Published:** 2019-01-01

**Authors:** Cecilia M. Casadei, Karol Nass, Anton Barty, Mark S. Hunter, Celestino Padeste, Ching-Ju Tsai, Sébastien Boutet, Marc Messerschmidt, Leonardo Sala, Garth J. Williams, Dmitry Ozerov, Matthew Coleman, Xiao-Dan Li, Matthias Frank, Bill Pedrini

**Affiliations:** a Paul Scherrer Institute, 5232 Villigen PSI, Switzerland; b Center for Free-Electron Laser Science, DESY, Notkestrasse 85, 22607 Hamburg, Germany; c Lawrence Livermore National Laboratory, 7000 East Avenue, Livermore, CA 94550, USA; d Linac Coherent Light Source, 2575 Sand Hill Road, Menlo Park, CA 94025, USA; e National Science Foundation BioXFEL Science and Technology Center, 700 Ellicott Street, Buffalo, NY 14203, USA; f NSLS-II, Brookhaven National Laboratory, PO Box 5000, Upton, NY 11973, USA

**Keywords:** free-electron lasers, serial femto­second crystallography, membrane proteins, two-dimensional crystals

## Abstract

Serial femtosecond crystallography of two-dimensional membrane-protein crystals at X-ray free-electron lasers has the potential to address the dynamics of functionally relevant large-scale motions, which can be sterically hindered in three-dimensional crystals and suppressed in cryocooled samples. In previous work, diffraction data limited to a two-dimensional reciprocal-space slice were evaluated and it was demonstrated that the low intensity of the diffraction signal can be overcome by collecting highly redundant data, thus enhancing the achievable resolution. Here, the application of a newly developed method to analyze diffraction data covering three reciprocal-space dimensions, extracting the reciprocal-space map of the structure-factor amplitudes, is presented. Despite the low resolution and completeness of the data set, it is shown by molecular replacement that the reconstructed amplitudes carry meaningful structural information. Therefore, it appears that these intrinsic limitations in resolution and completeness from two-dimensional crystal diffraction may be overcome by collecting highly redundant data along the three reciprocal-space axes, thus allowing the measurement of large-scale dynamics in pump–probe experiments.

## Abstract 2

Three-dimensional intensities were reconstructed from serial crystallography data using two-dimensional crystals.

## 1. Introduction   

The ultrashort and ultrabright pulses provided by hard X-ray free-electron lasers (XFELs) have enabled innovative experimental investigation methods to address new scientific problems. In the field of macromolecular crystallography, which traditionally uses three-dimensional crystals as samples, the femtosecond pulse duration on one hand allows most radiation damage to be outrun (Lomb *et al.*, 2011 ▸; Barty *et al.*, 2011 ▸; Nass *et al.*, 2015 ▸), making measurements at room temperature and/or with smaller and smaller crystals (to the submicrometre range) possible (Gati *et al.*, 2017 ▸). On the other hand, structural dynamics at 1–3 Å resolution that can be triggered externally, such as side-chain movements or cofactor isomerizations, become accessible on the femtoscond time scale by pump–probe experiments (Kern *et al.*, 2014 ▸; Kupitz *et al.*, 2014 ▸; Tenboer *et al.*, 2014 ▸; Barends *et al.*, 2015 ▸; Nango *et al.*, 2016 ▸; Nogly *et al.*, 2016 ▸; Young *et al.*, 2016 ▸; Suga *et al.*, 2017 ▸) and on the millisecond timescale by mix-and-inject experiments (Stagno *et al.*, 2016 ▸; Olmos *et al.*, 2018 ▸). Because each crystal is destroyed by the interaction with the X-ray pulse, the single-shot diffraction data have to be recorded from a large number of crystals to achieve sufficient completeness and redundancy. This data-acquisition strategy is called serial femtosecond crystallography (SFX). The crystals are delivered to the beam within a running liquid or viscous jet (Chapman *et al.*, 2011 ▸; Boutet *et al.*, 2012 ▸; Weierstall *et al.*, 2014 ▸) or by a solid support that is scanned through the beam (Hunter *et al.*, 2014 ▸; Cohen *et al.*, 2014 ▸; Roedig *et al.*, 2017 ▸). From the data-analysis point of view, the main challenge to be solved was the merging of diffraction patterns from crystals of different sizes and in random orientations illuminated by X-ray pulses of variable intensity and wavelength (White *et al.*, 2012 ▸; Sauter *et al.*, 2013 ▸; Neutze *et al.*, 2015 ▸; Schlichting, 2015 ▸).

Conformational changes at larger length scales of 3–6 Å are more challenging for investigation with three-dimensional crystals because the motions may be sterically hindered (Kühlbrandt, 2000 ▸). If available, two-dimensional crystals represent an opportunity because of the looser intermolecular contacts owing to the single layer of molecules. Membrane proteins are definitely the most relevant candidates (Stahlberg *et al.*, 2001 ▸) because their function typically involves such structural modifications (see, for example, Subramaniam & Henderson, 2000 ▸) and because the arrangement in two dimensions more closely mimics the environment on the cell membrane. When the relevant structural modifications take place on submillisecond timescales, investigations by electron microscopy and electron diffraction on samples whose dynamics have been frozen by flash-cooling are difficult (Subramaniam & Henderson, 1999 ▸). This opens a niche for SFX on two-dimensional crystals in pump–probe mode.

The diffraction signal of two-dimensional crystals is markedly lower than for analogous three-dimensional crystals with the same dimensions because the diffracting volume is orders of magnitude smaller and the reciprocal-space region that generates the diffraction is not concentrated in Bragg points but is diluted over one-dimensional Bragg rods. Because of the weak signal and the ill-effects of radiation damage, measuring high-resolution diffraction data from two-dimensional protein crystals at a continuous X-ray source is extremely challenging. Data collection at an XFEL represents a viable alternative (Frank *et al.*, 2014 ▸; Pedrini *et al.*, 2014 ▸). In recent work, we showed that the signal-to-noise ratio of the diffracted intensities is substantially enhanced by summing equivalent portions of images across the data set (Casadei *et al.*, 2018 ▸). In this way, the resolution of a highly redundant data set collected in November 2013 from two-dimensional bacteriorhodopsin crystals at zero tilt angle, *i.e.* with the incoming X-ray beam perpendicular to the crystal plane, could be extended from about 6 Å to the detector edge at 4 Å.

With zero-tilt data only a reciprocal-space slice is sampled, corresponding to two points on each Bragg rod. During the same November 2013 beamtime, we also collected data at a few different nonzero tilt angles, which cover three dimensions in reciprocal space and led to a genuine three-dimensional data set. We report here on the application of a novel method to merge the diffraction images and determine the structure-factor amplitudes along the Bragg rods. These were then phased by molecular replacement. The composite OMIT maps (Terwilliger *et al.*, 2008 ▸) indicate that, despite their low completeness and the limited resolution of about 6 Å, the experimental data contain meaningful structural information. The measures required to improve the data quality, which are crucial to follow structural dynamics in future pump–probe experiments, are then discussed.

## 2. Results   

### 2.1. Bragg rod intensity reconstruction   

Sets of 1000 diffraction images of two-dimensional bacteriorhodopsin D96N mutant crystals were collected at the CXI experimental station of the LCLS free-electron laser at three different tilt angles η = 5, 15 and 20°. The diffraction images were analyzed assuming *p*3 symmetry (planar space group 13) of the crystal (Henderson *et al.*, 1990 ▸), with two unit-cell vectors of equal length *a* forming an angle γ of 120°. The corresponding two-dimensional reciprocal-space unit cell is spanned by two vectors of length 2π/*a* forming an angle of 60°. With the further assumption that Friedel symmetry of the diffraction intensity is valid, it follows that the point group of the structure-factor amplitudes, and thus of the diffracted intensity, is *S*_6_.

**Figure 1:**
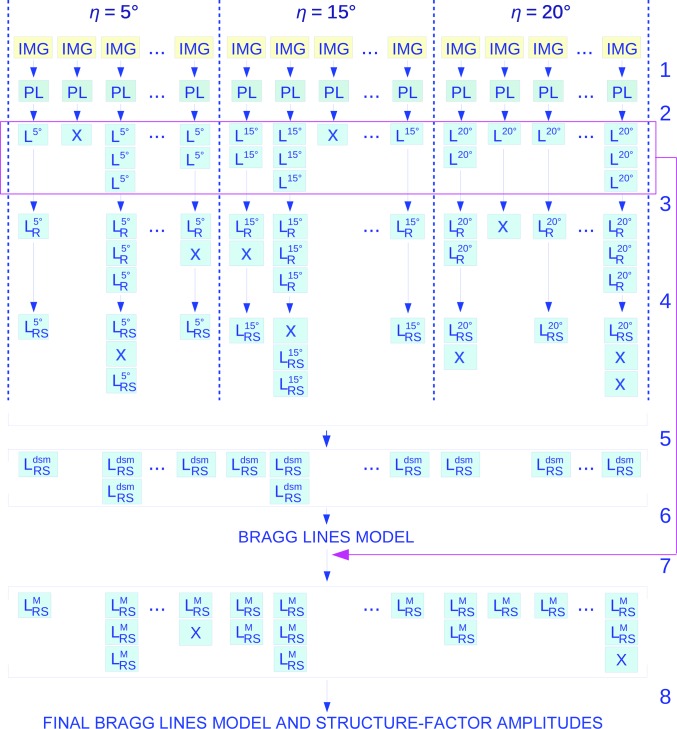
Data-analysis protocol. Abbreviations: IMG, diffraction image; PL, peak list; L, lattice; X, discarded; R, re-­indexed; S, scaled; dsm, after data-set merging; M, model. Step 1, extraction of a list of high-intensity peaks from each diffraction image. Step 2, lattice identification and refinement, spot search on the images, refinement of parameters and spot integration. Step 3, indexing-ambiguity solution within each data set. Step 4, scaling of intensities within each data set. Step 5, indexing-ambiguity solution and scaling between different data sets. Step 6, data merging and modeling of intensities along Bragg lines. Step 7, indexing-ambiguity solution and scaling of lattices determined in Step 2 using the intensity model as a reference. Step 8, data merging and fitting to obtain the final intensity model and structure-factor amplitude extraction.

The data-analysis pipeline to calculate the structure-factor amplitudes consists of eight subsequent steps, which are schematized in Fig. 1 ▸. Because it presents a number of novel aspects with respect to that applied previously to untilted data (Casadei *et al.*, 2018 ▸), the pipeline is outlined below in some detail and for each step the obtained outcome is mentioned explicitly.

**Figure 2:**
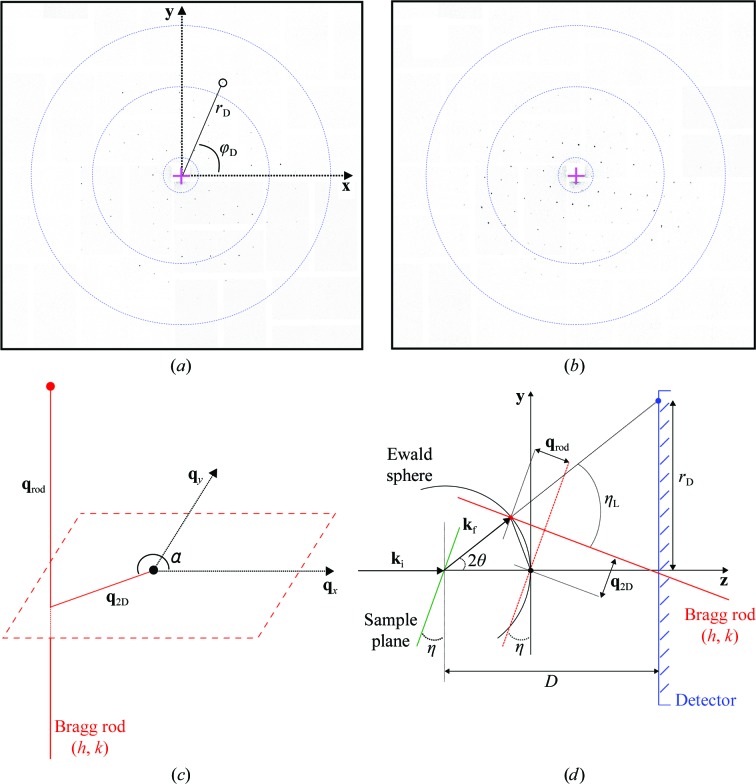
Diffraction from two-dimensional crystals. (*a*, *b*) Example diffraction images at 5° and 20° tilt angle, respectively. The circles are drawn at 50, 10 and 6 Å resolution. In (*a*), the detector-plane position in polar coordinates (*r*_D_, φ_D_) of a peak is superimposed. (*c*) Three-dimensional reciprocal space with a Bragg rod *h* a* + *k* b* + 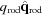 represented as a thick red line. (*d*) Ewald sphere construction for the diffraction process. The incoming and diffracted wavevectors are labeled **k**_i_ and **k**_f_, respectively. The diffracted beam is generated by the red dot on the Bragg rod. The diffraction angle of the Bragg peak on the detector (blue dot) has scattering angle 2θ. The sample plane with tilt angle η is represented in green.

**Figure 3:**
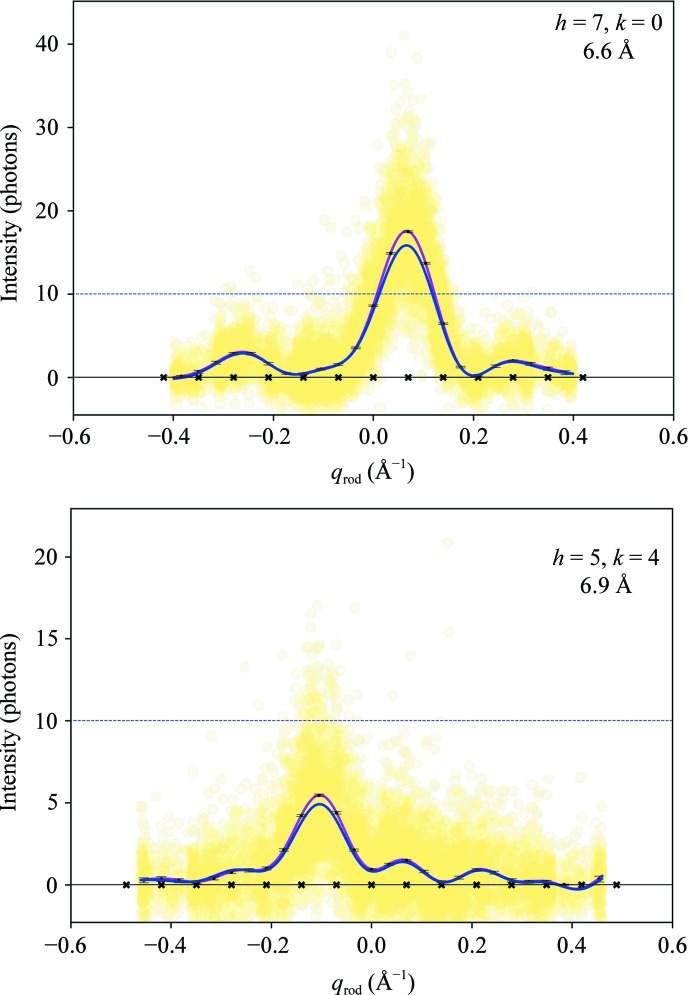
Bragg rod intensities. Intensity observations as a function of *q*_rod_ for Bragg rods {(7, 0)} and {(5, 4)}. Yellow dots, experimental observations. Blue and magenta lines, preliminary and final intensity model from (3); the black crosses along the horizontal axis mark the base points of the sinc functions of the model. Black dots with error bars, Bayesian estimates of intensities at discrete *q*_rod_ sampling points. The Miller indices *h*, *k* and the in-plane resolution of each Bragg rod are indicated. The dashed blue line represents the intensity level corresponding to ten photons.

*Step 1*. The software *Cheetah* (Barty *et al.*, 2014 ▸) was first employed to apply dark-current and gain corrections to the raw diffraction data. Two examples are shown in Figs. 2 ▸(*a*) and 2 ▸(*b*), which clearly show patterns from tilted, *p*3-symmetric two-dimensional crystals. The peaks are visible by eye down to a resolution of 7 Å. *Cheetah* was then used to generate a list of coordinates of the positions of the peaks, identified as clusters of pixels with high intensity.
*Step 2*. The peaks were grouped according to their mutual compatibility with a Bragg peak pattern from a single *p*3-symmetric two-dimensional crystal with lattice constant fixed at *a* = 62.45 Å (Henderson *et al.*, 1990 ▸) positioned on a plane at a given tilt angle η. A Bragg peak originates from the intersection of the Ewald sphere with a Bragg rod labeled by two integers (*h*, *k*) (Figs. 2 ▸ *c* and 2 ▸ *d*), and its position on the detector **r**_D,obs_, given in terms of the angles (θ, φ_D_), can be calculated as a function of (*h*, *k*, φ, η), where φ is the in-plane orientation angle parameter (see Section 4). Hence, imposing the best matching of the observed and the predicted peak positions on the detector gave a first estimate of φ. The grouping of the peaks and the optimization of φ were performed with an algorithm similar to that used for untilted data (Casadei *et al.*, 2018 ▸). If the peak subset associated with one two-dimensional crystal contained at least 18 *Cheetah* peaks, all potential Bragg peaks were searched directly in the experimental diffraction images as connected regions of high-intensity pixels in proximity to the positions predicted assuming the orientation φ. If at least 45% of the predicted peaks with in-plane resolution down to 6 Å were identified, the peak set was called ‘lattice’ and kept for the following evaluation steps. Up to four lattices were extracted from single images. The total number of lattices is reported in the third row of Table 1 ▸.For each lattice, the unit-cell parameter *a*, the in-plane crystal orientation φ and the direct beam coordinates *x_b_*, *y_b_* were further refined simultaneously by minimizing the expression 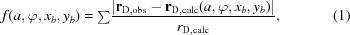
where the sum is over all identified peaks and **r**_D,calc_ is the predicted Bragg peak position on the detector. The minimum was determined by using multiple iterations of either a systematic grid search in the four-dimensional parameter space with progressively finer grid spacing or the Powell algorithm (Powell, 1964 ▸). The average refined unit-cell vector length was 62.62 Å, with a standard deviation of 0.06 Å. The shift of the beam position (Δ*x_b_* ^2^ + Δ*y_b_* ^2^)^1/2^ was typically below one pixel. We refrained from optimizing additional parameters such as the X-ray wavelength (energy) and the lattice tilt direction because of the large overhead in computational time.For each predicted peak position on a Bragg rod (*h*, *k*), the out-of-plane momentum transfer *q*_rod_(*h*, *k*, φ, η) was calculated (see Section 4), and the intensity *I*(*h*, *k*, *q*_rod_) was determined by integration in a circular area of radius corresponding to five detector pixels, after having subtracted a background modeled by an affine function. To account for the Lorentz and polarization effects, each intensity was multiplied by the correction factor 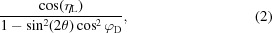
where η_L_ is the angle between the diffracted beam direction and the Bragg rod.
*Step 3*. Any transformation of the crystal inside the two-dimensional crystal plane which leaves the Bragg peak positions unaltered but is not a symmetry of the structure-factor amplitude leads to an ambiguity in indexing the lattice. Rotation of the crystal by 180° around the crystal-plane normal is always such a transformation, the ambiguity being in the assignment of a peak to either the reciprocal-space point *h*, *k*, *q*_rod_ or the point *h*, *k*, −*q*_rod_. Furthermore, in the considered *S*_6_ case, rotations of a crystal by multiples of 60° preserve the peak positions, but only rotations by multiples of 120° are symmetries of the structure-factor amplitude. Finally, the face-flip of the two-dimensional crystal also preserves the peak position but not the structure-factor amplitude, with the resulting ambiguity being between (*h*, *k*, *q*_rod_) and (*k*, *h*, −*q*_rod_). Because of the *S*_6_ symmetry, each reflection could be mapped for simplicity to an equivalent reflection on a Bragg rod (*h*, *k*) with *h* ≥ 0 and *k* > 0. With this simplification, the re-indexing of the lattice consists of transforming the indices assigned to each peak with one of the following operations, in order to achieve the best mutual correlation of intensities of equivalent peaks from pairs of lattices: 
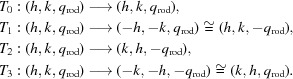
For the last two transformations the equivalence (0, *k*, *q*_rod_) ≡ (*k*, 0, −*q*_rod_) is used to maintain the condition *k* > 0. The determination of the per-lattice transformations was performed similar to the case of untilted data (Casadei *et al.*, 2018 ▸; see Section 4), in which peaks with an in-plane resolution of 7 Å were considered. The success of the re-indexing was between 60% and 90% depending on the data set, as reported in the fourth row of Table 1 ▸.
*Step 4*. To account for the fluctuations of the crystal area illuminated by X-rays and of the intensity of the X-ray pulse itself, a lattice-dependent multiplicative factor was calculated to scale the intensities from diffraction patterns recorded at the same tilt angle. The rescaling factors were determined with a procedure generalizing that applied previously for untilted data (Casadei *et al.*, 2018 ▸; see Section 4), comparing intensities of equivalent peaks from different lattices. Two peaks from different lattices on a same rod {(*h*, *k*)} were considered to be comparable if the difference in *q*_rod_ was smaller than a threshold value of 0.003 Å^−1^. Bragg rods down to a three-dimensional resolution of 7 Å were considered. As shown in the fifth row of Table 1 ▸, the rescaling factor could be determined for 50–75% of the lattices, depending on the data set.
*Step 5*. The three data sets collected at different tilt angles were merged into a single set by data-set-specific re-indexing, which was performed similarly to the procedure in Step 3 to maximize intensity correlations between the sets, and overall rescaling by data-set-specific multiplicative factors, which was performed in analogy to Step 4 to obtain the best correspondence of the intensities.
*Step 6*. The intensities of each Bragg rod (*h*, *k*) were modeled using the Levenberg–Marquardt algorithm for least-squares minimization with the function 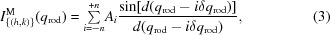
with fit parameters *A_i_*, which was proposed to handle analog electron-diffraction data from two-dimensional crystals (Crowther *et al.*, 1970 ▸; Leifer & Henderson, 1983 ▸; Baldwin & Henderson, 1984 ▸). It consists of a sum of sinc functions centered at equidistant base points with spacing δ*q*_rod_ = 2π/2*d*, defined by Shannon’s sampling theorem (Shannon, 1949 ▸) for a two-dimensional crystal of thickness *d* in the out-of-plane direction (see Appendix *A* [App appa]).
*Step 7*. All lattices, including those rejected previously, were again re-indexed and rescaled but now using the model *I* ^M^_{(*h*,*k*)}_(*q*_rod_) obtained in Step 6 as a reference. The re-indexing transformation of each lattice was set as that giving the best correlation for the linear regression between the lattice intensities and the corresponding intensities of the model. Only those lattices for which the correlation coefficient exceeded 0.92 were retained, and the rescaling factor was then set as the regression coefficient. With the mentioned threshold, about 50–80% of the initial lattices were retained (depending on the data set), as shown in row 6 of Table 1 ▸. The rationale behind this additional iteration was to cross-validate the model and retain as many lattices as possible for the next steps. At this point the data set consisted of Bragg rods {(*h*, *k*)}, each comprising about 9000 intensity observations labeled *I*′_{(*h*,*k*)}_(*q*_rod_) and measured at different out-of-plane momenta *q*_rod_. Each intensity measurement is shown as a yellow dot in the example Bragg rods in Fig. 3 ▸ and Supplementary Fig. S1.
*Step 8*. For each Bragg rod, the intensities *I*′_{(*h*,*k*)}_ were fitted again with the model function of (3) to obtain an updated intensity model *I* ^M,′^_{(*h*,*k*)}_(*q*_rod_), represented by the magenta line in the examples in Fig. 3 ▸ and Supplementary Fig. S1. The new model is almost identical to the first model, which is shown in blue in the figure.In view of molecular replacement, the intensities *I*′_{(*h*,*k*)}_(*l*) were extracted at discrete, equally spaced points *q*_rod_ = *l*δ*q*_rod_, with the spacing δ*q*_rod_ = (1/2)(2π/2*d*) corresponding to oversampling by a factor of two with respect to the minimal Shannon sampling interval. The error associated with each intensity value was calculated by propagating the errors on the fit parameters *A_i_* in (3). Finally, the intensities and the errors were corrected using the procedure devised by French & Wilson (1978 ▸), which is routinely used in three-dimensional protein crystallography to handle meaningless negative intensities derived from the experimental data. The corrected intensities *I* ^FW^_{(*h*,*k*)}_(*l*) are shown as black points in Fig. 3 ▸ with the corrected error bars. As expected, only the lower intensities are subject to a relevant correction. The French and Wilson procedure also provides corrected values of the structure-factor amplitudes *F* ^o^_{(*h*,*k*)}_(*l*) and their error estimates, the calculated values of which are reported in the Supporting Information. Table 2 ▸ summarizes the data-set statistics as typically reported in traditional three-dimensional crystallo­graphy. The quality indicators in the last five columns were calculated as described in Section 4, with some modifications with respect to the three-dimensional case to account for the different structure of the two-dimensional SFX data. Their values indicate that data to the detector edge, corresponding to 6 Å in-plane resolution, can be retained for further analysis. Obviously, the data suffer from the same missing cone problem as in electron diffraction (Unwin & Henderson, 1975 ▸), which gives a very low completeness of 38.2% inside the resolution sphere at 5.3 Å.

### 2.2. Density maps from molecular replacement   

Although the data set was of low completeness, the experimental structure-factor amplitudes *F* ^o^_{(*h*,*k*)}_(*l*) and the corresponding error estimates were used as input for molecular replacement and subsequent rigid-body refinement (see Section 4). The bacteriorhodopsin structure obtained by electron microscopy and diffraction, available as entry 1fbb in the Protein Data Bank (Berman *et al.*, 2003 ▸), was used as a starting model.

**Figure 4:**
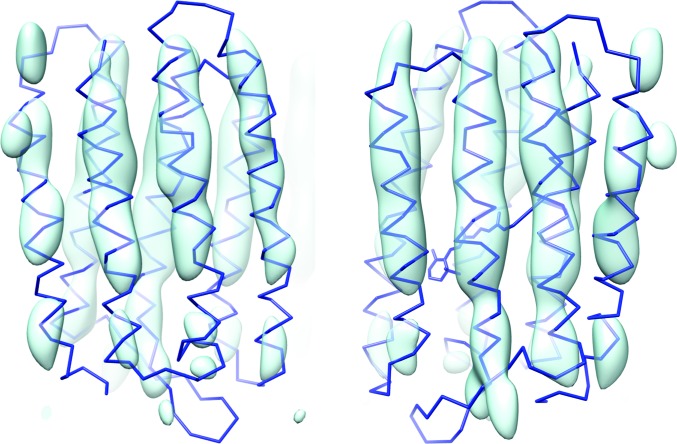
Composite OMIT map. Blue trace, backbone of atomic model 1fbb rigid-body refined using the experimental data with sampling δ*q*_rod_ = (1/2)(π/*d*). Cyan surface, the composite OMIT electron-density map with a contour level of 3.0σ. The structure is shown from two different views.

Owing to the bias introduced by the use of model phases, molecular-replacement maps are not to be considered representative of the information content of the data. To assess this content, we calculated the composite OMIT map (see Section 4), which is shown in Fig. 4 ▸ for two different views of the molecule. The map shows that the data contain information about the position and orientation of the α-helices in the structure. Although the presence of a missing cone of data in the *q*_rod_ direction leads to real-space features which are elongated along the *z* axis, at sufficiently high contour levels only density overlapping with the expected positions of helices is present in the maps. These conclusions also emerge from the ‘single-helices’ OMIT maps (see Section 4) shown in Supplementary Fig. S3. Furthermore, the findings are reproduced by the molecular-replacement procedure from the intensities sampled at δ*q*_rod_ = 2π/2*d* and δ*q*_rod_ = (1/4)2π/2*d* (see Supplementary Figs. S4 and S5).

## 3. Discussion   

We present a protocol which allowed a three-dimensional X-ray diffraction data set from two-dimensional protein crystals to be analysed. The three-dimensionality of the data set in reciprocal space is a consequence of the tilting of the membrane supporting the samples with respect to the X-ray beam. These data differ from electron diffraction data in that the Ewald sphere cannot be considered to be flat, even at low resolution, and in that each lattice diffraction pattern is a snapshot from one crystal, independent of any other pattern and not, for example, a representative in a tilt series from the same crystal. These differences triggered the development of a novel method which combines approaches from traditional X-ray crystallography (lattice identification, lattice-parameter refinement and Bayesian estimates of unique intensities, amplitudes and their error), three-dimensional SFX (merging of images from individual crystals affected by indexing ambiguity and intensity scaling) and two-dimensional electron diffraction (intensity modeling along Bragg rods). Applying this method, we reconstructed the diffraction intensities along Bragg rods in reciprocal space, from which the structure-factor amplitudes were extracted and their phases were determined by molecular replacement. The electron-density composite OMIT maps show that despite their low completeness and resolution, the data are meaningful.

The completeness is enhanced by recording data sets at higher tilt angles, which in general increases the *q*_rod_ coverage of the Bragg rods (see Supplementary Fig. S2). As a concrete example, with a tilt of 40° the completeness for the same crystal structure down to the same resolution range increases to 68.9%. Increasing the tilt angle unfortunately leads to an increased background because of the longer path of the X-ray beam inside the sample support. The image-summing approach presented in previous work (Casadei *et al.*, 2018 ▸), which aims to extend the achievable resolution by enhancing the signal-to-noise ratio, can be generalized straightforwardly. Obviously, to achieve the same improvement, for each *q*_rod_ bin of a tilted data set the same redundancy as an untilted data set has to be achieved, boosting the amount of required sample and the data-collection time by orders of magnitude. In this regard, the new high-speed scanning stage that has recently been commissioned at the CXI station opens new perspectives (Roedig *et al.*, 2017 ▸).

**Figure 5:**
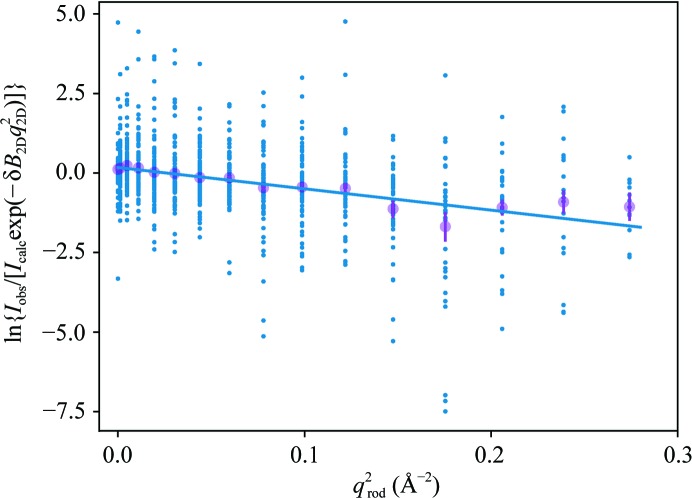
Anisotropic intensity decay. Blue dots, ln{*I*_obs_/[*I*_calc_exp(−δ*B*_2D_ *q*_2D_^2^)]}, with δ*B*_2D_ = −0.27 Å^2^, as a function of *q* ^2^_rod_. Magenta dots, bin averages with error bars. Blue line, curve ln*A* − δ*B*_rod_ *q* ^2^_rod_ with δ*B*_rod_ = 6.70 Å^2^.

In agreement with the results from pioneering two-dimensional electron diffraction work (Unwin & Henderson, 1975 ▸), we observe that the intensities decay in an anisotropic fashion with increasing resolution. We quantify this effect by modeling the ratio |*F* ^o^|^2^/|*F* ^m,iso^|^2^, with model amplitudes *F* ^m,iso^ calculated in the same way as above from the 1fbb model but without anisotropic *B* factors (see Section 4), with a two-dimensional Gaussian function:
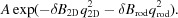
The fit is shown in Fig. 5 ▸, and the values of the obtained fit parameters δ*B*_2D_ = −0.27 Å^2^ and δ*B*_rod_ = 6.70 Å^2^ indicated that the experimental intensities decay remarkably faster in the *q*_rod_ direction than in the in-plane direction. We carried out the same treatment using observed and model structure factors from PDB entry 5b6v (Nango *et al.*, 2016 ▸), a structure of bR from three-dimensional SFX. In this case the fit parameters were refined to δ*B*_2D_ = 0.39 Å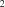 and δ*B*_rod_ = 0.51 Å^2^, showing that anisotropic effects are negligible with three-dimensional crystals. The large decay rate of two-dimensional crystal intensities along *q*_rod_ hints at increased disorder in the real-space out-of-plane direction as expected for a single-layer arrangement. Such an increase can be quantified by observing that the difference between experimental and model mean-square out-of-plane displacements amounts to approximately 6.7 Å^2^.

In conclusion, we have shown that the structure-factor amplitudes derived from the two-dimensional SFX data contain meaningful and structural information, and have made the point that the completeness and resolution limitations are overcome by enhancing the redundancy in the data collection. It therefore appears that with the present status of XFELs, three-dimensional difference electron-density maps at a few ångströms resolution can be determined between protein molecules with different configurations in two-dimensional crystals. Of particular interest are large-scale configuration changes on this length scale that are sterically hindered in three-dimensional crystals. If these movements are triggered by optical stimuli, two-dimensional SFX data sets can be measured at different delays between the exciting laser pulse and the X-ray probing pulse, in a fashion that is nowadays standard in three-dimensional SFX (Standfuss & Spence, 2017 ▸).

## 4. Methods   

### 4.1. Sample preparation   

Purple membrane was isolated from *Halobacterium salinarum* expressing the gene for the D96N bacteriorhodopsin mutant (bR-D96N) and detergent-stabilized two-dimensional crystal suspensions were prepared using previously described procedures (Frank *et al.*, 2014 ▸; Pedrini *et al.*, 2014 ▸). The two-dimensional crystals were washed with 6 m*M* octylglucoside, suspended in 0.5%(*w*/*v*) glucose to a final protein concentration of 0.4 mg ml^−1^ and subsequently applied onto the sample carrier for X-ray diffraction data collection.

Silicon chips with areas of 25 × 25 and 12.5 × 25 mm^2^ with 200 µm thickness, produced by Silson Inc., were used as sample carriers. The chips had a 44 × 44 or 22 × 44 array of 100 × 100 µm windows of 20 nm thick Si_3_N_4_. A total of about 20 µl bR-D96N two-dimensional crystal suspension was deposited onto the silicon chip and allowed to dry in air. The resulting glucose layer served to protect the protein sample from dehydration.

### 4.2. Experimental setup and data collection   

The X-ray diffraction measurements were carried out using the 0.1 µm focus setup of the CXI experimental station (Liang *et al.*, 2015 ▸) at the Linac Coherent Light Source. The beam size was estimated to be below 200 nm full width at half maximum (FWHM). The photon energy was set to 8.5 keV (1.5 Å), the pulse energy was approximately 2 mJ and the pulse length was approximately 35 fs FWHM.

The chips covered with two-dimensional bR-D96N crystals were mounted on a metallic frame that was fixed to the sample stages inside the vacuum experimental chamber. The sample stages were scanned in steps at a rate of about 1.5 s^−1^. The silicon frames were kept in a nonperpendicular configuration with respect to the X-ray beam, with tilt angles of 5, 15 and 20° about the *x* axis (Fig. 2 ▸). Diffraction patterns were recorded using a 2.3 megapixel Cornell–SLAC pixel-array detector, which was positioned 285 mm downstream of the sample in the same vacuum chamber (Blaj *et al.*, 2015 ▸).

### 4.3. Software   

Unless specified otherwise, the processing was performed using dedicated algorithms written in the Python 2.7 language, which are available on request.

### 4.4. Peak indexing   

The geometry of the diffraction experiment using two-dimensional crystals is schematized in Figs. 2 ▸(*c*) and 2 ▸(*d*), where *z* denotes the direction of the incoming X-ray beam and η denotes the sample-support tilt angle about the *x* axis. The reciprocal-space plane spanned by the reciprocal basis vectors a* and b* can alternatively be described using the orthonormal vectors 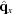 and 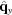, with 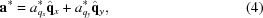


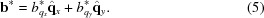
The in-plane component of the momentum-transfer vector **q** = **k**_f_ − **k**_i_ is
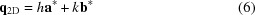
and forms an azimuthal angle α with the 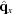 axis given by 
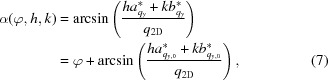
where φ is the random in-plane orientation of the two-dimensional crystal and 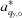 and 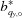 are the components of a* and b* along 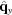 when the two-dimensional crystal is in the reference in-plane orientation. The transferred wavevector is 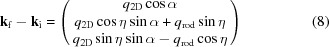
with
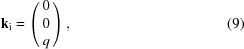
where *q* = 2π/λ. By squaring **k**_f_ and solving for *q*_rod_ one obtains 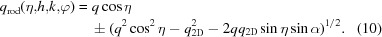
By considering 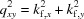
and replacing with the values from (8), the following expression for the azimuth detector coordinate of the diffraction spot is obtained,
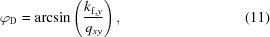
where
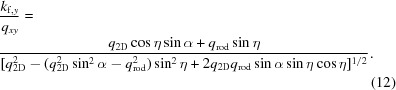
The radial coordinate of the diffraction spot is 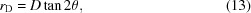
where *D* is the detector distance and 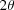 is the scattering angle given by 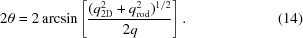



### 2.5. Re-indexing   

The task is determining lattice-specific re-indexing transformations **T_i_** (with *i* = 0, 1, 2, 3) that make the assignment of reciprocal-space indices coherent across the data set. The set of possible transformations is
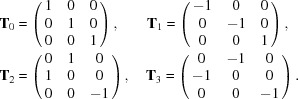
A lattice is randomly extracted from the data set and used as a reference *R*. The transformation **T**_*LR*_ required to re-index lattice *L* and make it compatible with *R* is established based on the calculation of intensity correlation coefficients. This determination of **T**_*LR*_ is accepted if the expression
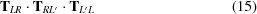
equals the identity for at least 70% of a large number (∼100) of randomly selected lattices *L*′. The procedure is repeated using different reference lattices and the consistency of the results is checked.

The transformation 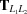 required to re-index *L*_1_ and make it compatible with *L*_2_ is determined by calculating a correlation coefficient between intensities from the two lattices in each of the four different indexing scenarios. The largest coefficient is considered to be representative of the correct transformation. The intensity correlation coefficient CC_*i*_ related to the transformation **T**_i_ is calculated by matching every spot (*h*, *k*, *q*_rod_) in *L*_1_ to any of the following *p*3-symmetry equivalent spots in *L*_2_, 
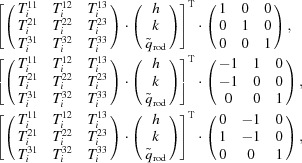
where T indicates the transpose, and their Friedel mates. Importantly, in the general tilt case it is necessary to allow 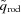 ≃ *q*_rod_ and to set an upper limit on the absolute *q*_rod_ difference of matched spots.

### 2.6. Evaluation of data quality   

A merging residual was calculated in three-dimensional resolution bins, 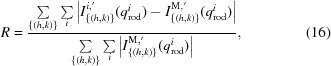
where the sum extends over all Bragg lines {(*h*, *k*)} and observations *i* within the resolution bin (Baldwin & Henderson, 1984 ▸). A half-data-set correlation coefficient CC_1/2_ was calculated per resolution bin as follows. Observations in each *q*_rod_ bin of width π/(2*d*) were split randomly into two groups of (approximately) equal size and the linear correlation coefficient between half-data-set averages was calculated. The random splitting was repeated ten times and the average correlation coefficient was considered. The value of CC* was calculated according to the definition in Karplus & Diederichs (2012 ▸). The global CC_1/2_ and CC* values were calculated as the weighted averages of individual bin values, with weights based on the number of unique reflections. Signal-to-noise ratios were calculated as three-dimensional resolution-bin averages of ratios between Bayesian estimates of intensities and their standard deviations for unique reflections. Completeness values in three-dimensional resolution bins were calculated as the ratio between the number of reciprocal-space points sampled by two-dimensional crystal diffraction with tilt angle 20°, considering δ*q*_rod_ = π/2*d*, and the number of points within the corresponding spherical shell.

### 2.7. Molecular replacement   

Bayesian estimates of unique reflection structure-factor amplitudes *F* ^o^ and their errors were converted to MTZ format using the *CCP*4 program *F*2*MTZ* (Winn *et al.*, 2011 ▸). The data were phased by molecular replacement in *Phaser* (McCoy *et al.*, 2007 ▸) using the structural model 1fbb (Subramaniam & Henderson, 2000 ▸) from the Protein Data Bank (Berman *et al.*, 2003 ▸). The solution was rigid-body refined in *PHENIX* (Adams *et al.*, 2010 ▸) to obtain model structure-factor amplitudes and phases (*F* ^c^, φ^c^). To verify that the model was not biased by the phases from molecular replacement, we calculated composite OMIT electron-density maps (Terwilliger *et al.*, 2008 ▸) using the *PHENIX* software suite. We also carried out the standard procedure of removing a portion of the model, in this case a sequence of 20 amino acids, and using structure-factor amplitudes and phases (*F* ^c,OMIT^, φ^c,OMIT^) determined from the truncation of the complete model. The Fourier coefficients of the OMIT maps (*mF* ^o^ − *DF* ^c,OMIT^)exp(*i*φ^c,OMIT^), where *m* are the Sim weights and *D* are the Luzzati factors (Read, 1986 ▸), are conceived so that any feature accounted for in the data, but absent in the model, is represented by a region of positive electron density in the map.

### 2.8. Anisotropy modeling   

To estimate anisotropic decay parameters, model structure factors *F* ^m,iso^ were calculated using *PHENIX* (Adams *et al.*, 2010 ▸) with disabled anisotropic scaling and including bulk-solvent corrections.

## Supplementary Material

Supplementary Figures and Table.. DOI: 10.1107/S2052252518014641/ec5011sup1.pdf

## Figures and Tables

**Table 1:** Summarizing table The results of data processing including success rates of indexing-ambiguity solution and scale-factor determination as explained in the text.

η (data-set tilt angle)	5°	15°	20°
No. of collected images	968	1232	968
Percentage of images with lattices	58.2%	63.4%	58.2%
No. of lattices	992 (100%)	1383 (100%)	799 (100%)
No. of re-indexed lattices (first)	902 (90.9%)	1180 (85.3%)	507 (63.4%)
No. of re-indexed and rescaled lattices (first)	771 (77.7%)	1027 (74.3%)	419 (52.4%)
No. of re-indexed and rescaled lattices (second)	513 (51.7%)	1020 (73.8%)	651 (81.5%)

**Table 2:** Data-processing statistics in three-dimensional resolution bins Columns 1 and 2, resolution range; column 3, number of observations; column 4, number of unique reflections; column 5, ratio of the two previous columns; columns 6–10, merging *R* value, half-data-set correlation coefficient (CC_1/2_), CC*, signal-to-noise ratio (S/N) and completeness as defined in Section 4.

Low (Å)	High (Å)	*N*_obs_	*N*_unique_	*N*_obs_/*N*_unique_	*R*	CC_1/2_	CC*	S/N	Completeness
54.09	25.20	32141	13	2472.38	0.18	0.9999	1.0000	138.69	0.459
25.20	15.61	32619	24	1359.12	0.38	0.9991	0.9998	71.93	0.310
15.61	12.71	31643	39	811.36	0.26	0.9995	0.9999	77.77	0.398
12.71	11.63	32228	34	947.88	0.27	0.9994	0.9998	93.58	0.513
11.63	10.18	32579	44	740.43	0.22	0.9997	0.9999	77.11	0.347
10.18	9.40	31688	54	586.81	0.29	0.9989	0.9997	58.76	0.463
9.40	8.64	31915	55	580.27	0.22	0.9996	0.9999	89.70	0.378
8.64	7.93	32254	86	375.05	0.47	0.9968	0.9992	27.38	0.451
7.93	7.52	32138	58	554.10	0.43	0.9985	0.9996	48.39	0.396
7.52	7.13	32036	76	421.53	0.56	0.9977	0.9994	27.80	0.421
7.13	6.79	33074	86	384.58	0.76	0.9921	0.9980	17.79	0.477
6.79	6.54	30574	75	407.65	0.81	0.9898	0.9974	20.09	0.448
6.54	6.20	33199	109	304.58	1.13	0.9778	0.9944	12.39	0.423
6.20	6.02	31883	86	370.73	1.16	0.9679	0.9918	14.37	0.494
6.02	5.30	31985	151	211.82	2.55	0.8233	0.9503	4.09	0.238
54.09	5.30	481956	990	486.82	0.30	0.9657	0.9906	35.11	0.382

## Appendix A Derivation of the intensity model

We show that the summation in (3) represents an appropriate model for intensities in the *q*_rod_ direction because of the finite thickness *d* of the molecule monolayer. The diffracted intensity is the square amplitude of the Fourier transform of the charge density,

This is equivalent to the expression

*P* is the charge-density autocorrelation, *q_z_* is the reciprocal-space coordinate along the direction of the Bragg lines, *x* and *y* are fractional coordinates in terms of the real-space crystallographic vectors **a** and **b**, and *z* is the real-space coordinate in the direction perpendicular to the plane spanned by **a** and **b**. It should be emphasized that since the monolayer has a finite thickness *d*, the extent of the autocorrelation function is limited to |*z*| ≤ *d*. The autocorrelation function can be expressed by means of a discrete sum

where the sampling points {*q*_*z*,*i*_} along Bragg lines are equally spaced by 1/(2*d*) as defined by Shannon’s sampling theorem. Including (19) in (18) gives

Considering that

and

the following expression for intensity is obtained,

leading to

The integral term in (24) is the Fourier transform of a step function extending from −*d* to +*d*, namely a sinc function

(25) corresponds to (3), where the factor 2π is included in the definition of *q*_rod_.
