# Supplementary material for: Structure-factor amplitude reconstruction from serial femtosecond crystallography of two-dimensional membrane-protein crystals
Source: IUCrJ. 2019 Jan 1;6(Pt 1):34–45. doi: 10.1107/S2052252518014641 (PMC6327180; doi:10.1107/S2052252518014641)
Supplement: Supplementary file 1 [file m-06-00034-sup1.pdf]

IUCrJ

Volume 6 (2019)

Supporting information for article:

Structure-factor amplitude reconstruction from serial femtosecond crystallography of two-dimensional membrane-protein crystals

Cecilia M. Casadei, Karol Nass, Anton Barty, Mark S. Hunter, Celestino Padeste, Ching-Ju Tsai, Sébastien Boutet, Marc Messerschmidt, Leonardo Sala, Garth J. Williams, Dmitry Ozerov, Matthew Coleman, Xiao-Dan Li, Matthias Frank and Bill Pedrini

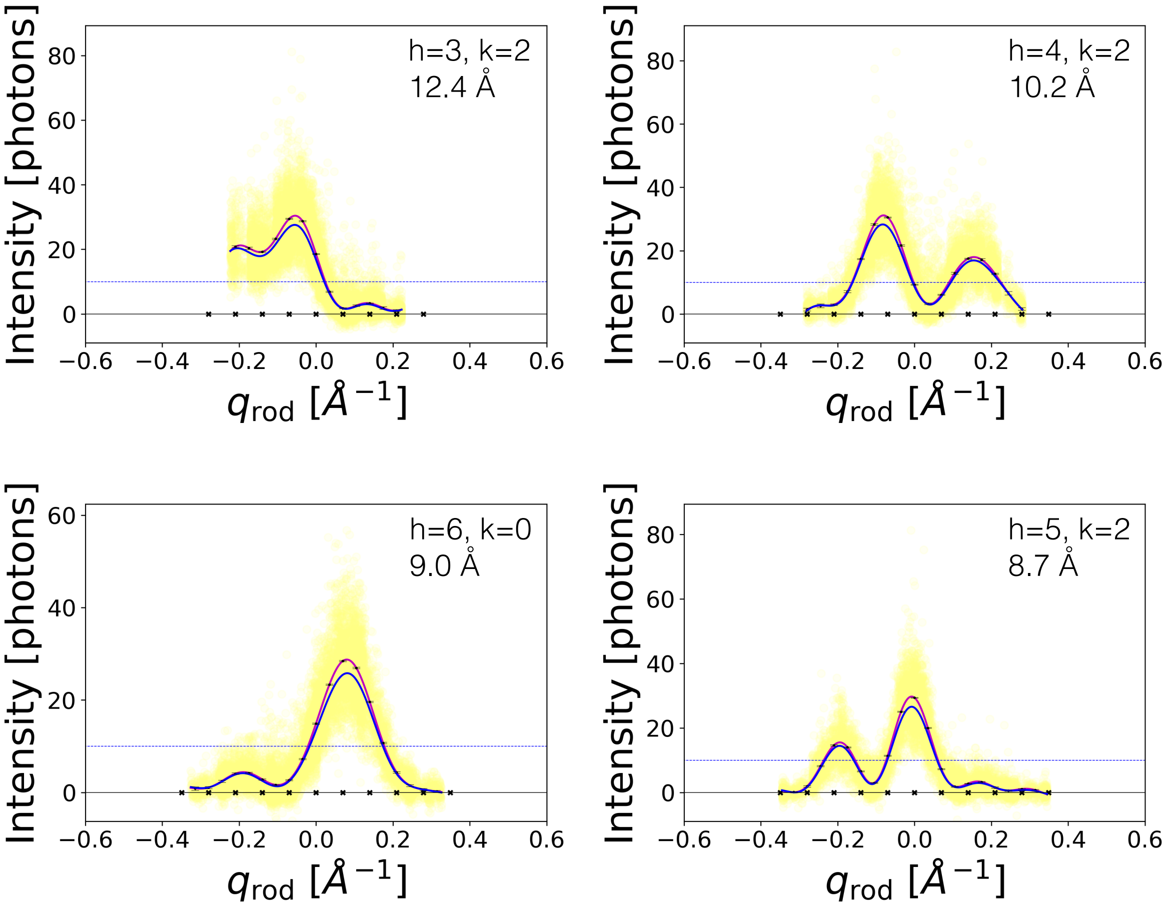

Fig. S1. *Bragg rod intensities.* Intensity observations as a function of q_{rod} for several Bragg rods. Yellow dots: experimental observations. Blue and magenta lines: preliminary and final intensity model from Equation 3; the black crosses along the horizontal axis mark the base points of the sinc functions of the model. Black dots with error bars: bayesian estimates of intensities at discrete q_{rod} sampling points. The Miller indices h, k and the in-plane resolution of each Bragg rod are indicated. The dashed blue line represents the intensity level corresponding to 10 photons.

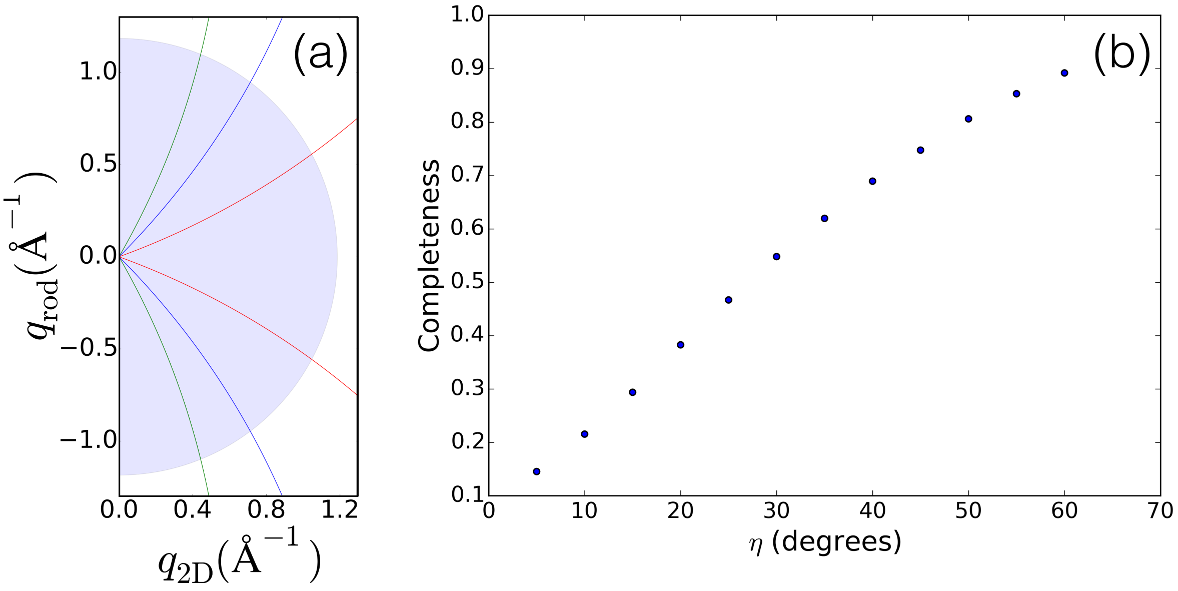

Fig. S2. *Reciprocal space coverage.* (a) Extension of the reciprocal space sampling along q_{rod} as a function of q_{2D} for different tilt angles. Red: 20°, blue: 45°, green: 60°. The resolution sphere at 5.3 Å is represented. (b) Fraction of sampled reciprocal space, assuming a three dimensional resolution limit of 5.3 Å, as a function of the tilt angle.

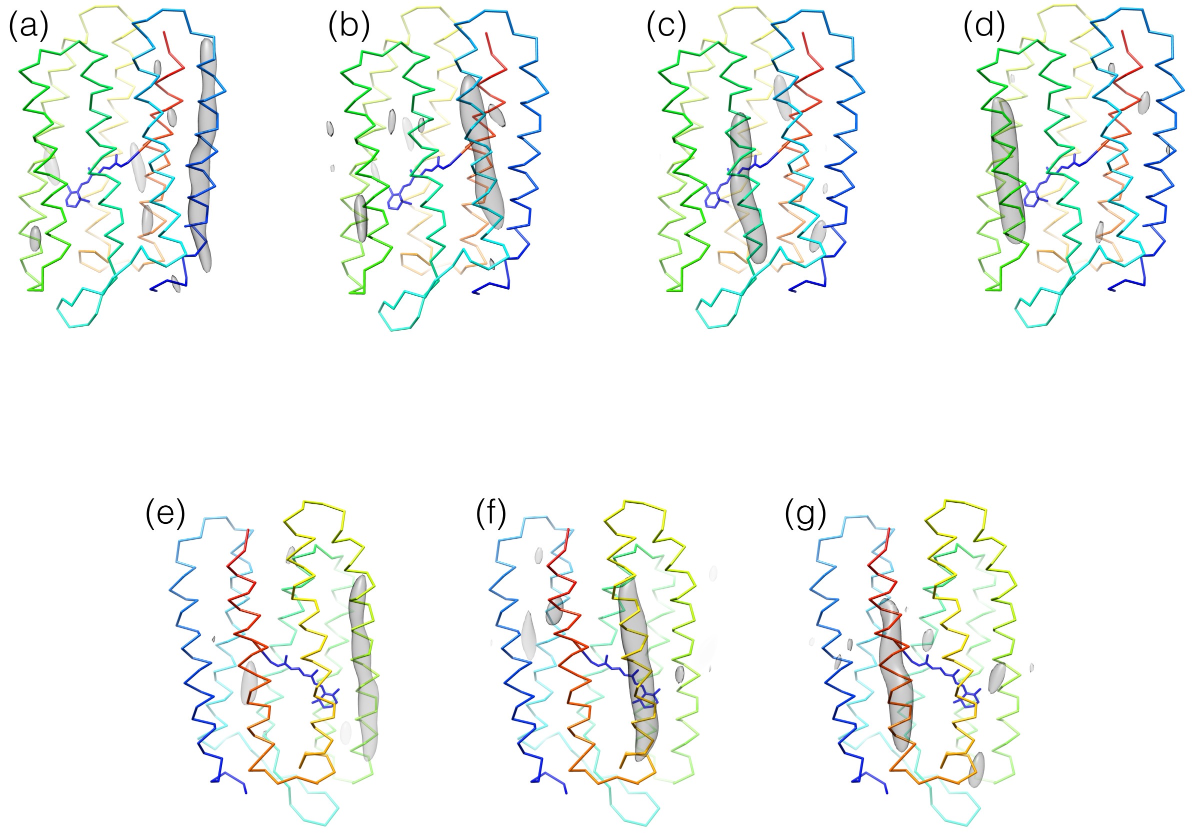

Fig. S3. *Electron density omit maps from incomplete models.* Rainbow traces: backbone of the atomic model 1FBB after rigid body refinement. Grey surfaces: omit electron density maps obtained by removing sequences of 20 aminoacid from the model. The map is contoured at 5.0σ. (a) Residues 11 - 30 were omitted, (b) 42 - 61, (c) 78 - 97, (d) 109 - 128, (e) 135 - 154, (f) 172 - 191, (g) 200 - 219. Experimental intensities were sampled along Bragg lines with $\delta q_{\mathrm{rod}} = \frac{1}{2}\frac{\pi}{d}$.

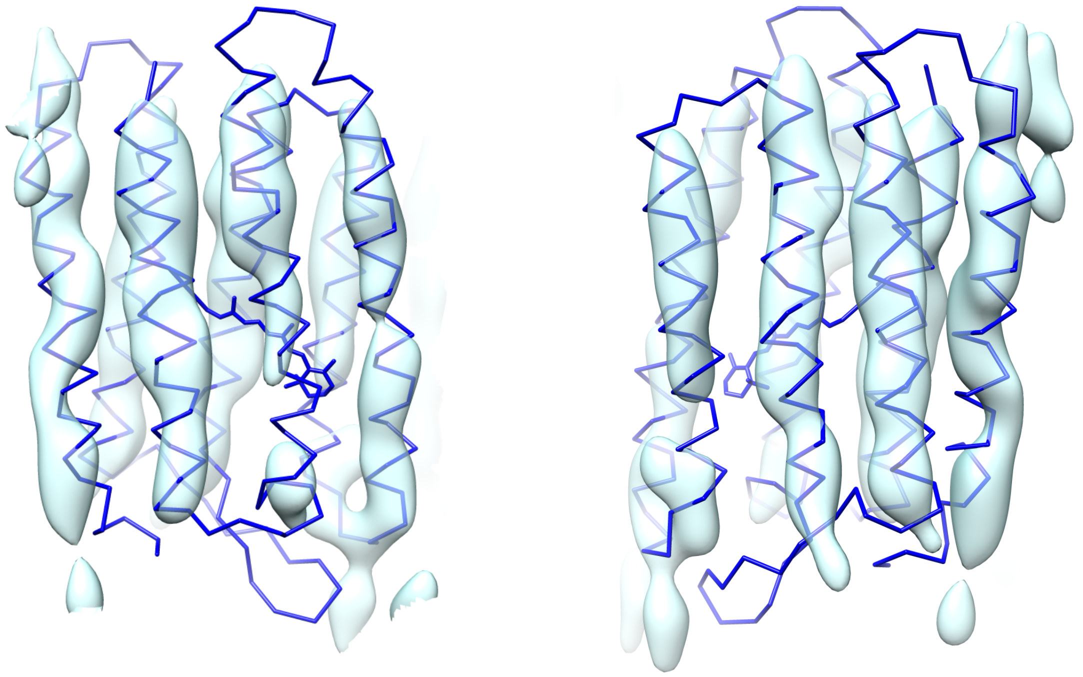

Fig. S4. *Composite omit map.* Blue trace: backbone of the atomic model 1FBB, rigid body refined using the experimental data with sampling $\delta q_{\mathrm{rod}} = \frac{\pi}{d}$. Cyan surface: contour level of the composite omit electron density map with contour level of 2.0σ. The structure is shown from two different views.

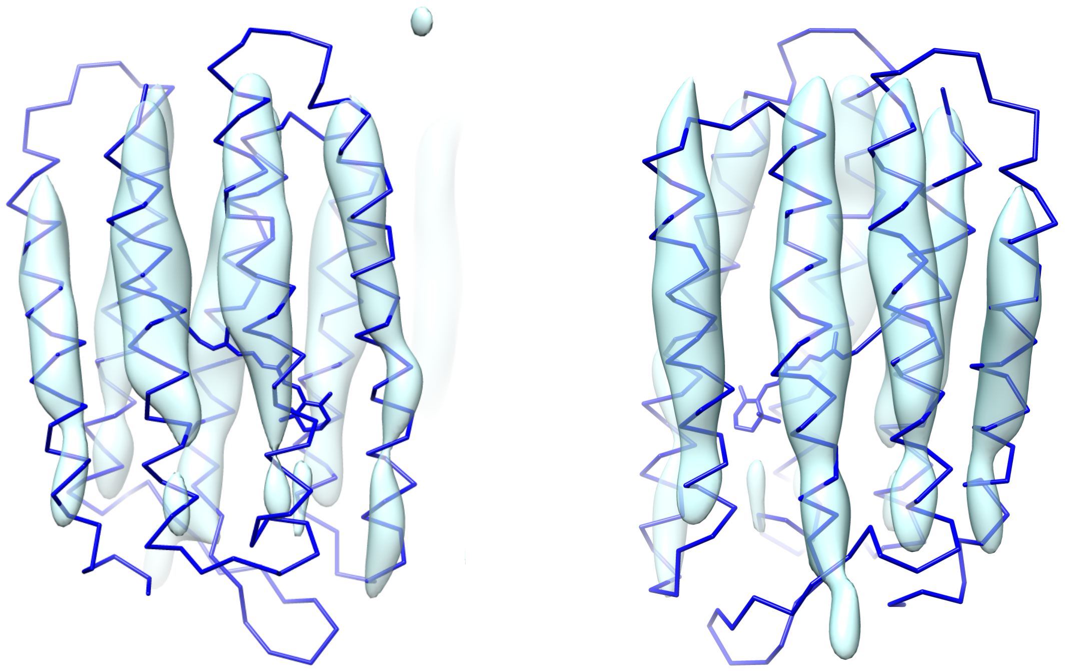

Fig. S5. *Composite omit map.* Blue trace: backbone of the atomic model 1FBB, rigid body refined using the experimental data with sampling $\delta q_{\mathrm{rod}} = \frac{1}{4}\frac{\pi}{d}$. Cyan surface: contour level of the composite omit electron density map with contour level of 5.0σ. The structure is shown from two different views.

*List of structure factor amplitudes

Table S1: List of structure factor amplitudes (number of photons) and their error as obtained using the French and Wilson approach.

h	k	l	F^o	$\sigma(F^o)$
	0	-1	2.26	0.02
1	0	0	1.94	0.02
1	0	1	3.34	0.02
1	1	-2	7.51	0.02
1	1	-1	6.22	0.02
1	1	0	7.20	0.01
1	1	1	9.29	0.01
1	1	2	10.27	0.02
2	0	-2	6.37	0.02
2	0	-1	5.62	0.01
2	0	0	5.09	0.01
2	0	1	5.03	0.01
2	0	2	5.02	0.02
1	2	-3	2.77	0.02
1	2	-2	3.12	0.02
1	2	-1	3.16	0.02
1	2	0	3.32	0.01
1	2	1	3.70	0.02
1	2	2	3.83	0.02
1	2	3	3.34	0.02
2	1	-3	2.35	0.02
2	1	-2	2.79	0.02
2	1	-1	2.65	0.02
2	1	0	2.08	0.02
2	1	1	1.48	0.03
2	1	2	1.23	0.03
2	1	3	1.13	0.04
3	0	-4	2.70	0.03
3	0	-3	2.38	0.02
3	0	-2	1.98	0.02
3	0	-1	1.91	0.02
3	0	0	2.07	0.01
3	0	1	2.09	0.02
3	0	2	1.89	0.02
3	0	3	1.75	0.02
3	0	4	1.83	0.03
2	2	0	4.58	0.02

Continuation of Table 1

h	k	l	F^o	$\sigma(F^o)$
2	2	-4	0.94	0.06
2	2	-3	1.67	0.02
2	2	-2	2.71	0.02
2	2	-1	3.76	0.02
2	2	1	4.98	0.02
2	2	2	4.83	0.02
2	2	3	4.10	0.02
2	2	4	2.96	0.04
1	3	-5	0.74	0.07
1	3	-4	0.61	0.08
1	3	-3	1.11	0.04
1	3	-2	1.63	0.04
1	3	-1	1.91	0.04
1	3	0	1.98	0.03
1	3	1	1.87	0.05
1	3	2	1.57	0.04
1	3	3	1.08	0.05
1	3	4	0.62	0.07
1	3	5	0.73	0.07
3	1	-5	1.08	0.06
3	1	-4	1.58	0.04
3	1	-3	2.51	0.02
3	1	-2	3.87	0.02
3	1	-1	5.19	0.03
3	1	0	5.98	0.02
3	1	1	5.94	0.03
3	1	2	5.13	0.02
3	1	3	3.89	0.02
3	1	4	2.71	0.03
3	1	5	1.92	0.04
4	0	-4	3.45	0.02
4	0	-3	4.48	0.02
4	0	-2	5.02	0.02
4	0	-1	5.07	0.02
4	0	0	4.71	0.02
4	0	1	4.09	0.02
4	0	2	3.37	0.02
4	0	3	2.71	0.02
4	0	4	2.16	0.02
4	0	-5	2.06	0.04
4	0	5	1.65	0.04

Continuation of Table 1

h	k	l	F^o	$\sigma(F^o)$
2	3	-5	2.43	0.03
2	3	-4	3.41	0.03
2	3	-3	4.15	0.02
2	3	-2	4.37	0.02
2	3	-1	3.93	0.02
2	3	0	2.89	0.02
2	3	1	1.57	0.04
2	3	2	1.24	0.04
2	3	3	2.10	0.03
2	3	4	2.64	0.03
2	3	5	2.76	0.03
3	2	-5	4.51	0.03
3	2	-4	4.39	0.03
3	2	-3	4.83	0.02
3	2	-2	5.43	0.02
3	2	-1	5.36	0.02
3	2	0	4.31	0.02
3	2	1	2.62	0.03
3	2	2	1.34	0.04
3	2	3	1.61	0.04
3	2	4	1.82	0.03
3	2	5	1.36	0.04
4	1	-2	3.59	0.02
4	1	-1	4.91	0.02
4	1	0	5.34	0.02
4	1	1	4.79	0.02
4	1	2	3.52	0.02
1	4	-2	1.43	0.03
1	4	-1	2.78	0.03
1	4	0	4.51	0.02
1	4	1	5.46	0.02
1	4	2	5.27	0.02
2	3	-6	1.66	0.04
2	3	6	2.83	0.04
3	2	-6	4.56	0.05
3	2	6	1.02	0.08
4	1	-6	2.75	0.05
4	1	-5	2.00	0.03
4	1	-4	0.91	0.04
4	1	-3	1.80	0.03
4	1	3	2.01	0.03

Continuation of Table 1

h	k	l	F^o	$\sigma(F^o)$
4	1	4	0.84	0.05
4	1	5	0.98	0.04
4	1	6	1.67	0.05
1	4	-6	2.57	0.04
1	4	-5	2.77	0.02
1	4	-4	2.78	0.03
1	4	-3	2.10	0.02
1	4	3	4.11	0.02
1	4	4	2.50	0.02
1	4	5	1.25	0.04
1	4	6	1.18	0.07
5	0	-5	2.53	0.03
5	0	-4	3.07	0.03
5	0	-3	3.46	0.02
5	0	-2	4.41	0.02
5	0	-1	5.96	0.02
5	0	0	7.35	0.02
5	0	1	7.91	0.02
5	0	2	7.41	0.02
5	0	3	6.02	0.02
5	0	4	4.18	0.03
5	0	5	2.39	0.03
3	3	-3	0.83	0.04
3	3	-2	0.67	0.05
3	3	-1	0.85	0.04
3	3	0	1.16	0.03
3	3	1	1.72	0.03
3	3	2	2.38	0.02
3	3	3	2.84	0.02
4	2	-1	4.65	0.03
4	2	0	3.05	0.03
4	2	1	1.79	0.05
2	4	-1	7.46	0.03
2	4	0	8.16	0.02
2	4	1	7.43	0.03
5	0	-7	1.14	0.05
5	0	-6	1.67	0.04
5	0	6	1.14	0.05
5	0	7	0.97	0.05
3	3	-7	2.65	0.06
3	3	-6	2.64	0.03

Continuation of Table 1

h	k	l	F^o	$\sigma(F^o)$
3	3	-5	2.19	0.03
3	3	-4	1.48	0.04
3	3	4	2.92	0.03
3	3	5	2.68	0.03
3	3	6	2.36	0.03
3	3	7	2.13	0.06
4	2	-7	1.61	0.08
4	2	-6	1.72	0.03
4	2	-5	2.66	0.03
4	2	-4	4.17	0.03
4	2	-3	5.32	0.02
4	2	-2	5.53	0.02
4	2	2	2.46	0.03
4	2	3	3.59	0.02
4	2	4	4.18	0.03
4	2	5	4.14	0.03
4	2	6	3.56	0.03
4	2	7	2.56	0.07
2	4	-7	1.75	0.05
2	4	-6	1.53	0.03
2	4	-5	2.13	0.03
2	4	-4	3.06	0.04
2	4	-3	4.19	0.02
2	4	-2	5.83	0.02
2	4	2	5.57	0.02
2	4	3	3.42	0.02
2	4	4	1.86	0.05
2	4	5	0.98	0.05
2	4	6	0.81	0.06
2	4	7	1.37	0.06
5	1	-4	4.60	0.04
5	1	-3	4.10	0.02
5	1	-2	3.00	0.02
5	1	-1	1.91	0.03
5	1	0	1.29	0.03
5	1	1	1.42	0.04
5	1	2	2.39	0.03
5	1	3	3.56	0.02
5	1	4	4.26	0.04
1	5	-4	1.70	0.06
1	5	-3	0.93	0.04

Continuation of Table 1

h	k	l	F^o	$\sigma(F^o)$
1	5	-2	2.19	0.03
1	5	-1	4.10	0.03
1	5	0	5.22	0.02
1	5	1	5.00	0.03
1	5	2	3.66	0.02
1	5	3	2.39	0.02
1	5	4	2.72	0.04
4	2	-8	1.20	0.06
4	2	8	1.27	0.05
2	4	-8	0.83	0.09
2	4	8	0.72	0.09
5	1	-8	0.63	0.08
5	1	-7	1.66	0.04
5	1	-6	3.04	0.03
5	1	-5	4.20	0.03
5	1	5	4.13	0.03
5	1	6	3.29	0.03
5	1	7	2.17	0.05
5	1	8	1.11	0.05
1	5	-8	1.81	0.04
1	5	-7	2.14	0.04
1	5	-6	2.08	0.04
1	5	-5	2.08	0.03
1	5	5	3.32	0.03
1	5	6	2.96	0.03
1	5	7	1.77	0.05
1	5	8	0.65	0.07
6	0	-5	2.06	0.03
6	0	-4	1.68	0.05
6	0	-3	1.27	0.03
6	0	-2	1.64	0.04
6	0	-1	2.69	0.03
6	0	0	3.86	0.02
6	0	1	4.83	0.03
6	0	2	5.33	0.02
6	0	3	5.19	0.02
6	0	4	4.43	0.04
6	0	5	3.27	0.03
4	3	-4	6.30	0.04
4	3	-3	8.02	0.02
4	3	-2	8.99	0.04

Continuation of Table 1

h	k	l	F^o	$\sigma(F^o)$
4	3	-1	9.08	0.03
4	3	0	8.68	0.02
4	3	1	8.18	0.03
4	3	2	7.54	0.03
4	3	3	6.46	0.02
4	3	4	4.98	0.04
3	4	-4	2.17	0.05
3	4	-3	3.27	0.03
3	4	-2	4.40	0.03
3	4	-1	5.28	0.03
3	4	0	5.63	0.02
3	4	1	5.39	0.03
3	4	2	4.73	0.04
3	4	3	3.95	0.02
3	4	4	3.24	0.04
5	2	-1	5.00	0.03
5	2	0	5.42	0.02
5	2	1	4.47	0.03
2	5	-1	1.83	0.05
2	5	0	2.06	0.03
2	5	1	2.23	0.04
6	0	-9	0.94	0.06
6	0	-8	1.02	0.09
6	0	-7	1.57	0.03
6	0	-6	2.02	0.04
6	0	6	2.09	0.04
6	0	7	1.23	0.04
6	0	8	0.78	0.09
6	0	9	0.44	0.13
4	3	-9	0.65	0.11
4	3	-8	0.03	0.10
4	3	-7	1.50	0.03
4	3	-6	2.78	0.04
4	3	-5	4.36	0.04
4	3	5	3.57	0.04
4	3	6	2.58	0.03
4	3	7	1.64	0.03
4	3	8	0.27	0.17
4	3	9	0.83	0.09
3	4	-9	0.25	0.13
3	4	-8	0.63	0.11

Continuation of Table 1

h	k	l	F^o	$\sigma(F^o)$
3	4	-7	0.48	0.07
3	4	-6	0.55	0.10
3	4	-5	1.25	0.05
3	4	5	2.61	0.03
3	4	6	2.06	0.04
3	4	7	1.68	0.04
3	4	8	1.38	0.07
3	4	9	0.86	0.09
5	2	-9	0.23	0.15
5	2	-8	1.38	0.08
5	2	-7	2.86	0.03
5	2	-6	3.82	0.04
5	2	-5	3.74	0.04
5	2	-4	2.56	0.03
5	2	-3	1.72	0.03
5	2	-2	3.37	0.03
5	2	2	2.70	0.03
5	2	3	1.35	0.03
5	2	4	1.66	0.03
5	2	5	1.80	0.03
5	2	6	1.25	0.06
5	2	7	0.76	0.07
5	2	8	1.03	0.08
5	2	9	0.85	0.09
2	5	-9	0.80	0.10
2	5	-8	0.70	0.09
2	5	-7	0.89	0.06
2	5	-6	1.38	0.06
2	5	-5	1.82	0.04
2	5	-4	2.00	0.03
2	5	-3	1.92	0.02
2	5	-2	1.78	0.04
2	5	2	2.28	0.03
2	5	3	2.34	0.02
2	5	4	2.48	0.03
2	5	5	2.58	0.03
2	5	6	2.49	0.04
2	5	7	2.18	0.03
2	5	8	1.75	0.06
2	5	9	1.36	0.07
6	1	-6	3.03	0.04

Continuation of Table 1

h	k	l	F^o	$\sigma(F^o)$
6	1	-5	3.26	0.04
6	1	-4	3.18	0.03
6	1	-3	3.21	0.02
6	1	-2	3.59	0.04
6	1	-1	3.91	0.03
6	1	0	3.73	0.02
6	1	1	3.05	0.03
6	1	2	2.28	0.04
6	1	3	2.01	0.03
6	1	4	2.08	0.03
6	1	5	1.91	0.04
6	1	6	1.37	0.05
1	6	-6	1.14	0.05
1	6	-5	1.20	0.05
1	6	-4	0.94	0.04
1	6	-3	0.56	0.07
1	6	-2	0.89	0.08
1	6	-1	1.40	0.04
1	6	0	1.53	0.03
1	6	1	1.27	0.04
1	6	2	1.13	0.05
1	6	3	1.45	0.03
1	6	4	1.72	0.03
1	6	5	1.62	0.04
1	6	6	1.27	0.04
6	1	-9	1.55	0.07
6	1	-8	1.84	0.04
6	1	-7	2.43	0.04
6	1	7	0.80	0.06
6	1	8	0.80	0.06
6	1	9	1.00	0.09
1	6	-9	0.58	0.12
1	6	-8	0.78	0.06
1	6	-7	0.94	0.07
1	6	7	1.03	0.05
1	6	8	0.97	0.05
1	6	9	0.71	0.10
4	4	-6	1.81	0.04
4	4	-5	2.44	0.05
4	4	-4	2.85	0.02
4	4	-3	2.86	0.03

Continuation of Table 1

h	k	l	F^o	$\sigma(F^o)$
4	4	-2	2.45	0.03
4	4	-1	1.84	0.03
4	4	0	1.52	0.03
4	4	1	1.78	0.03
4	4	2	2.19	0.03
4	4	3	2.44	0.02
4	4	4	2.45	0.02
4	4	5	2.18	0.05
4	4	6	1.61	0.03
5	3	-5	0.98	0.09
5	3	-4	0.82	0.04
5	3	-3	1.04	0.04
5	3	-2	1.34	0.05
5	3	-1	1.43	0.04
5	3	0	1.27	0.05
5	3	1	1.01	0.05
5	3	2	1.04	0.05
5	3	3	1.38	0.03
5	3	4	1.67	0.03
5	3	5	1.70	0.06
3	5	-5	1.18	0.07
3	5	-4	1.83	0.02
3	5	-3	2.62	0.02
3	5	-2	3.34	0.03
3	5	-1	3.90	0.03
3	5	0	4.30	0.03
3	5	1	4.45	0.03
3	5	2	4.23	0.03
3	5	3	3.60	0.02
3	5	4	2.67	0.02
3	5	5	1.69	0.05
7	0	-5	0.69	0.09
7	0	-4	0.66	0.05
7	0	-3	0.98	0.05
7	0	-2	1.24	0.04
7	0	-1	1.88	0.03
7	0	0	2.93	0.03
7	0	1	3.86	0.03
7	0	2	4.18	0.03
7	0	3	3.70	0.03
7	0	4	2.54	0.02

Continuation of Table 1

h	k	l	F^o	$\sigma(F^o)$
7	0	5	1.11	0.07
6	1	-10	1.18	0.05
6	1	10	0.89	0.05
1	6	-10	0.29	0.12
1	6	10	0.22	0.12
4	4	-10	0.11	0.15
4	4	-9	0.02	0.06
4	4	-8	0.51	0.10
4	4	-7	1.14	0.04
4	4	7	0.83	0.06
4	4	8	0.38	0.12
4	4	9	0.83	0.05
4	4	10	0.92	0.12
5	3	-9	1.40	0.04
5	3	-8	1.48	0.05
5	3	-7	1.45	0.05
5	3	-6	1.28	0.04
5	3	6	1.43	0.04
5	3	7	0.95	0.06
5	3	8	0.53	0.09
5	3	9	0.58	0.07
3	5	-9	0.98	0.04
3	5	-8	0.96	0.06
3	5	-7	0.96	0.07
3	5	-6	0.95	0.05
3	5	6	0.93	0.05
3	5	7	0.47	0.11
3	5	8	0.24	0.12
3	5	9	0.38	0.10
7	0	-9	1.33	0.04
7	0	-8	1.68	0.04
7	0	-7	1.68	0.05
7	0	-6	1.28	0.04
7	0	6	0.47	0.10
7	0	7	1.16	0.06
7	0	8	1.40	0.05
7	0	9	1.28	0.04
6	2	-7	0.54	0.12
6	2	-6	0.39	0.10
6	2	-5	0.40	0.12
6	2	-4	0.51	0.06

Continuation of Table 1

h	k	l	F^o	$\sigma(F^o)$
6	2	-3	0.51	0.08
6	2	-2	0.70	0.05
6	2	-1	1.17	0.04
6	2	0	1.63	0.03
6	2	1	1.86	0.03
6	2	2	1.77	0.03
6	2	3	1.38	0.03
6	2	4	0.83	0.04
6	2	5	0.30	0.11
6	2	6	0.36	0.11
6	2	7	0.64	0.08
2	6	-7	0.33	0.14
2	6	-6	0.20	0.11
2	6	-5	0.22	0.12
2	6	-4	0.80	0.04
2	6	-3	1.32	0.03
2	6	-2	1.70	0.03
2	6	-1	1.98	0.03
2	6	0	2.29	0.02
2	6	1	2.60	0.03
2	6	2	2.73	0.03
2	6	3	2.53	0.03
2	6	4	2.01	0.02
2	6	5	1.36	0.04
2	6	6	0.82	0.06
2	6	7	0.50	0.11
1	7	-2	3.96	0.03
1	7	-1	4.07	0.03
1	7	0	3.80	0.03
1	7	1	3.34	0.03
1	7	2	2.86	0.03
7	1	-2	1.91	0.03
7	1	-1	2.28	0.03
7	1	0	2.68	0.03
7	1	1	2.94	0.03
7	1	2	2.82	0.03
4	4	-11	0.23	0.11
4	4	11	0.24	0.11
5	3	-11	0.86	0.06
5	3	-10	1.19	0.09
5	3	10	0.66	0.14

Continuation of Table 1

h	k	l	F^o	$\sigma(F^o)$
5	3	11	0.51	0.09
3	5	-11	0.94	0.05
3	5	-10	1.02	0.09
3	5	10	0.45	0.17
3	5	11	0.27	0.11
7	0	-11	0.26	0.11
7	0	-10	0.80	0.11
7	0	10	1.03	0.09
7	0	11	0.77	0.05
6	2	-11	0.17	0.13
6	2	-10	0.19	0.15
6	2	-9	0.49	0.06
6	2	-8	0.62	0.08
6	2	8	0.78	0.07
6	2	9	0.79	0.05
6	2	10	0.65	0.14
6	2	11	0.47	0.13
2	6	-11	0.24	0.12
2	6	-10	0.30	0.14
2	6	-9	0.24	0.09
2	6	-8	0.26	0.12
2	6	8	0.26	0.12
2	6	9	0.09	0.10
2	6	10	0.10	0.13
2	6	11	0.03	0.08
1	7	-8	0.31	0.12
1	7	-7	0.53	0.13
1	7	-6	0.98	0.06
1	7	-5	1.72	0.03
1	7	-4	2.61	0.02
1	7	-3	3.44	0.03
1	7	3	2.43	0.03
1	7	4	1.97	0.03
1	7	5	1.46	0.03
1	7	6	1.05	0.05
1	7	7	1.06	0.07
1	7	8	1.35	0.06
7	1	-8	0.54	0.10
7	1	-7	0.64	0.10
7	1	-6	0.60	0.08
7	1	-5	0.67	0.06

Continuation of Table 1

h	k	l	F^o	$\sigma(F^o)$
7	1	-4	1.07	0.03
7	1	-3	1.53	0.03
7	1	3	2.29	0.02
7	1	4	1.55	0.03
7	1	5	1.13	0.04
7	1	6	1.27	0.04
7	1	7	1.38	0.06
7	1	8	1.21	0.07
5	4	-5	1.46	0.03
5	4	-4	2.05	0.03
5	4	-3	2.33	0.03
5	4	-2	2.09	0.03
5	4	-1	1.45	0.03
5	4	0	0.96	0.05
5	4	1	1.11	0.03
5	4	2	1.22	0.04
5	4	3	0.90	0.04
5	4	4	0.40	0.10
5	4	5	0.66	0.05
4	5	-5	1.78	0.03
4	5	-4	1.65	0.03
4	5	-3	1.32	0.03
4	5	-2	1.07	0.04
4	5	-1	1.23	0.03
4	5	0	1.62	0.04
4	5	1	1.91	0.03
4	5	2	1.96	0.03
4	5	3	1.75	0.03
4	5	4	1.40	0.03
4	5	5	1.15	0.04
6	3	-2	0.96	0.05
6	3	-1	1.00	0.04
6	3	0	0.91	0.06
6	3	1	1.06	0.04
6	3	2	1.47	0.04
3	6	-2	1.49	0.04
3	6	-1	1.48	0.03
3	6	0	1.08	0.05
3	6	1	0.63	0.06
3	6	2	0.70	0.06
1	7	-11	0.17	0.13

Continuation of Table 1

h	k	l	F^o	$\sigma(F^o)$
1	7	-10	0.20	0.11
1	7	-9	0.22	0.12
1	7	9	1.52	0.05
1	7	10	1.46	0.04
1	7	11	1.18	0.07
7	1	-11	0.64	0.12
7	1	-10	0.61	0.06
7	1	-9	0.46	0.10
7	1	9	0.92	0.06
7	1	10	0.67	0.07
7	1	11	0.46	0.14
5	4	-9	0.62	0.09
5	4	-8	0.88	0.07
5	4	-7	0.93	0.08
5	4	-6	0.99	0.06
5	4	6	0.95	0.05
5	4	7	0.85	0.08
5	4	8	0.51	0.11
5	4	9	0.41	0.11
4	5	-9	0.28	0.12
4	5	-8	0.50	0.12
4	5	-7	1.08	0.07
4	5	-6	1.57	0.04
4	5	6	1.07	0.05
4	5	7	1.04	0.06
4	5	8	0.92	0.08
4	5	9	0.78	0.06
6	3	-7	0.66	0.08
6	3	-6	0.41	0.11
6	3	-5	0.15	0.10
6	3	-4	0.24	0.10
6	3	-3	0.68	0.05
6	3	3	1.82	0.03
6	3	4	1.96	0.03
6	3	5	1.92	0.03
6	3	6	1.80	0.04
6	3	7	1.61	0.05
3	6	-7	0.96	0.06
3	6	-6	0.91	0.05
3	6	-5	0.53	0.06
3	6	-4	0.47	0.07

Continuation of Table 1

h	k	l	F^o	$\sigma(F^o)$
3	6	-3	1.08	0.03
3	6	3	1.00	0.04
3	6	4	1.16	0.04
3	6	5	1.25	0.04
3	6	6	1.34	0.04
3	6	7	1.30	0.05
8	0	-6	1.13	0.04
8	0	-5	1.75	0.03
8	0	-4	2.15	0.03
8	0	-3	2.15	0.03
8	0	-2	1.78	0.03
8	0	-1	1.17	0.04
8	0	0	0.61	0.08
8	0	1	0.67	0.05
8	0	2	1.17	0.04
8	0	3	1.62	0.03
8	0	4	1.86	0.03
8	0	5	1.79	0.03
8	0	6	1.42	0.04
7	2	-3	2.19	0.03
7	2	-2	1.72	0.03
7	2	-1	1.12	0.04
7	2	0	0.78	0.08
7	2	1	0.99	0.04
7	2	2	1.36	0.04
7	2	3	1.63	0.03
2	7	-3	1.47	0.03
2	7	-2	1.51	0.04
2	7	-1	2.03	0.03
2	7	0	2.48	0.04
2	7	1	2.51	0.03
2	7	2	2.16	0.03
2	7	3	1.82	0.03
1	7	-12	0.13	0.12
1	7	12	0.85	0.08
7	1	-12	0.36	0.12
7	1	12	0.36	0.12
5	4	-12	0.57	0.13
5	4	-11	0.46	0.15
5	4	-10	0.39	0.09
5	4	10	0.42	0.10

Continuation of Table 1

h	k	l	F^o	$\sigma(F^o)$
5	4	11	0.04	0.10
5	4	12	0.02	0.07
4	5	-12	0.16	0.14
4	5	-11	0.21	0.14
4	5	-10	0.40	0.10
4	5	10	0.63	0.08
4	5	11	0.53	0.12
4	5	12	0.54	0.13
6	3	-12	0.73	0.11
6	3	-11	0.94	0.13
6	3	-10	1.16	0.04
6	3	-9	1.14	0.05
6	3	-8	0.92	0.08
6	3	8	1.26	0.07
6	3	9	0.77	0.06
6	3	10	0.38	0.10
6	3	11	0.53	0.15
6	3	12	0.74	0.09
3	6	-12	0.28	0.13
3	6	-11	0.36	0.15
3	6	-10	0.46	0.08
3	6	-9	0.49	0.11
3	6	-8	0.73	0.09
3	6	8	1.02	0.07
3	6	9	0.54	0.10
3	6	10	0.12	0.11
3	6	11	0.34	0.15
3	6	12	0.54	0.11
8	0	-11	1.14	0.05
8	0	-10	1.00	0.06
8	0	-9	0.88	0.07
8	0	-8	0.70	0.12
8	0	-7	0.67	0.09
8	0	7	0.92	0.07
8	0	8	0.51	0.12
8	0	9	0.38	0.13
8	0	10	0.36	0.11
8	0	11	0.31	0.12
7	2	-10	0.32	0.11
7	2	-9	0.56	0.10
7	2	-8	0.82	0.09

Continuation of Table 1

h	k	l	F^o	$\sigma(F^o)$
7	2	-7	1.09	0.07
7	2	-6	1.54	0.03
7	2	-5	2.03	0.03
7	2	-4	2.30	0.03
7	2	4	1.68	0.03
7	2	5	1.48	0.04
7	2	6	1.05	0.04
7	2	7	0.59	0.09
7	2	8	0.40	0.14
7	2	9	0.52	0.11
7	2	10	0.59	0.09
2	7	-10	0.54	0.08
2	7	-9	0.91	0.08
2	7	-8	1.14	0.08
2	7	-7	1.22	0.06
2	7	-6	1.40	0.04
2	7	-5	1.65	0.03
2	7	-4	1.68	0.03
2	7	4	1.80	0.03
2	7	5	1.90	0.03
2	7	6	1.88	0.04
2	7	7	1.69	0.05
2	7	8	1.39	0.08
2	7	9	1.02	0.06
2	7	10	0.63	0.08
1	8	-5	1.81	0.03
1	8	-4	1.80	0.03
1	8	-3	1.57	0.03
1	8	-2	1.20	0.04
1	8	-1	0.86	0.05
1	8	0	0.83	0.06
1	8	1	1.19	0.04
1	8	2	1.56	0.03
1	8	3	1.74	0.03
1	8	4	1.70	0.03
1	8	5	1.50	0.04
8	1	-5	0.49	0.09
8	1	-4	0.27	0.10
8	1	-3	0.29	0.10
8	1	-2	0.45	0.08
8	1	-1	0.37	0.09

Continuation of Table 1

h	k	l	F^o	$\sigma(F^o)$
8	1	0	0.16	0.12
8	1	1	0.55	0.07
8	1	2	1.01	0.04
8	1	3	1.25	0.04
8	1	4	1.18	0.03
8	1	5	0.91	0.05
5	5	-3	1.19	0.04
5	5	-2	1.24	0.04
5	5	-1	1.06	0.04
5	5	0	0.81	0.06
5	5	1	0.69	0.05
5	5	2	0.66	0.06
5	5	3	0.63	0.07
4	6	-1	1.00	0.04
4	6	0	0.91	0.06
4	6	1	1.07	0.04
6	4	-1	1.04	0.04
6	4	0	1.31	0.04
6	4	1	1.35	0.03
5	4	-13	0.53	0.10
5	4	13	0.61	0.08
4	5	-13	0.54	0.10
4	5	13	0.67	0.09
6	3	-13	0.72	0.07
6	3	13	0.65	0.08
3	6	-13	0.43	0.12
3	6	13	0.47	0.10
8	0	-13	1.05	0.07
8	0	-12	1.21	0.05
8	0	12	0.34	0.13
8	0	13	0.39	0.11
7	2	-12	0.79	0.10
7	2	-11	0.57	0.07
7	2	11	0.65	0.07
7	2	12	0.65	0.12
2	7	-12	0.60	0.11
2	7	-11	0.47	0.09
2	7	11	0.47	0.11
2	7	12	0.46	0.13
1	8	-9	0.13	0.14
1	8	-8	0.43	0.14

Continuation of Table 1

h	k	l	F^o	$\sigma(F^o)$
1	8	-7	1.03	0.06
1	8	-6	1.54	0.03
1	8	6	1.27	0.03
1	8	7	1.01	0.07
1	8	8	0.66	0.11
1	8	9	0.23	0.14
8	1	-9	0.16	0.13
8	1	-8	0.07	0.12
8	1	-7	0.25	0.12
8	1	-6	0.53	0.07
8	1	6	0.71	0.06
8	1	7	0.78	0.09
8	1	8	0.93	0.08
8	1	9	0.93	0.10
5	5	-7	0.55	0.11
5	5	-6	0.59	0.06
5	5	-5	0.67	0.07
5	5	-4	0.94	0.04
5	5	4	0.74	0.05
5	5	5	0.94	0.05
5	5	6	0.95	0.04
5	5	7	0.66	0.09
4	6	-7	1.07	0.06
4	6	-6	1.55	0.03
4	6	-5	1.83	0.03
4	6	-4	1.86	0.03
4	6	-3	1.66	0.04
4	6	-2	1.33	0.04
4	6	2	1.21	0.04
4	6	3	1.18	0.04
4	6	4	0.99	0.04
4	6	5	0.78	0.06
4	6	6	0.62	0.06
4	6	7	0.41	0.13
6	4	-7	1.07	0.06
6	4	-6	1.24	0.04
6	4	-5	1.26	0.04
6	4	-4	1.06	0.04
6	4	-3	0.73	0.05
6	4	-2	0.70	0.06
6	4	2	1.19	0.04

Continuation of Table 1

h	k	l	F^o	$\sigma(F^o)$
6	4	3	0.93	0.05
6	4	4	0.76	0.05
6	4	5	0.88	0.06
6	4	6	1.14	0.04
6	4	7	1.27	0.05
3	7	-4	1.19	0.04
3	7	-3	1.53	0.04
3	7	-2	1.68	0.03
3	7	-1	1.57	0.03
3	7	0	1.25	0.05
3	7	1	0.96	0.04
3	7	2	0.95	0.04
3	7	3	1.16	0.04
3	7	4	1.39	0.03
7	3	-4	1.45	0.03
7	3	-3	1.67	0.04
7	3	-2	1.50	0.03
7	3	-1	1.15	0.04
7	3	0	1.20	0.04
7	3	1	1.58	0.03
7	3	2	1.77	0.03
7	3	3	1.57	0.03
7	3	4	1.09	0.04
7	2	-14	0.31	0.13
7	2	-13	0.71	0.10
7	2	13	0.43	0.15
7	2	14	0.04	0.09
2	7	-14	0.28	0.14
2	7	-13	0.36	0.14
2	7	13	0.31	0.16
2	7	14	0.62	0.10
1	8	-14	0.64	0.11
1	8	-13	0.53	0.13
1	8	-12	0.07	0.11
1	8	-11	0.00	0.02
1	8	-10	0.04	0.08
1	8	10	0.14	0.13
1	8	11	0.38	0.11
1	8	12	0.41	0.12
1	8	13	0.23	0.14
1	8	14	0.08	0.12

Continuation of Table 1

h	k	l	F^o	$\sigma(F^o)$
8	1	-14	0.53	0.14
8	1	-13	0.34	0.14
8	1	-12	0.08	0.11
8	1	-11	0.13	0.11
8	1	-10	0.27	0.12
8	1	10	0.73	0.08
8	1	11	0.32	0.11
8	1	12	0.02	0.07
8	1	13	0.12	0.14
8	1	14	0.53	0.13
5	5	-15	0.68	0.12
5	5	-14	0.64	0.14
5	5	-13	0.31	0.17
5	5	-12	0.08	0.11
5	5	-11	0.06	0.09
5	5	-10	0.11	0.12
5	5	-9	0.16	0.13
5	5	-8	0.34	0.13
5	5	8	0.13	0.13
5	5	9	0.27	0.13
5	5	10	0.64	0.11
5	5	11	0.70	0.06
5	5	12	0.46	0.13
5	5	13	0.25	0.17
5	5	14	0.35	0.14
5	5	15	0.18	0.13
4	6	-15	0.35	0.12
4	6	-14	0.45	0.15
4	6	-13	0.56	0.18
4	6	-12	0.83	0.06
4	6	-11	0.85	0.06
4	6	-10	0.63	0.11
4	6	-9	0.26	0.15
4	6	-8	0.45	0.14
4	6	8	0.22	0.13
4	6	9	0.48	0.13
4	6	10	0.93	0.07
4	6	11	1.19	0.06
4	6	12	1.21	0.05
4	6	13	0.90	0.15
4	6	14	0.59	0.13

Continuation of Table 1

h	k	l	F^o	$\sigma(F^o)$
4	6	15	0.30	0.13
6	4	-15	0.92	0.08
6	4	-14	1.03	0.09
6	4	-13	0.81	0.16
6	4	-12	0.48	0.11
6	4	-11	0.10	0.11
6	4	-10	0.37	0.13
6	4	-9	0.70	0.10
6	4	-8	0.88	0.08
6	4	8	1.16	0.07
6	4	9	0.86	0.08
6	4	10	0.50	0.12
6	4	11	0.37	0.11
6	4	12	0.32	0.13
6	4	13	0.15	0.17
6	4	14	0.10	0.14
6	4	15	0.27	0.13
3	7	-15	0.53	0.12
3	7	-14	0.32	0.14
3	7	-13	0.08	0.14
3	7	-12	0.07	0.09
3	7	-11	0.40	0.12
3	7	-10	0.48	0.13
3	7	-9	0.30	0.15
3	7	-8	0.08	0.12
3	7	-7	0.08	0.12
3	7	-6	0.38	0.10
3	7	-5	0.77	0.06
3	7	5	1.50	0.04
3	7	6	1.46	0.03
3	7	7	1.18	0.06
3	7	8	0.79	0.10
3	7	9	0.57	0.12
3	7	10	0.69	0.10
3	7	11	0.77	0.07
3	7	12	0.53	0.08
3	7	13	0.07	0.15
3	7	14	0.12	0.13
3	7	15	0.54	0.14
7	3	-15	0.06	0.11
7	3	-14	0.32	0.15

Continuation of Table 1

h	k	l	F^o	$\sigma(F^o)$
7	3	-13	0.17	0.22
7	3	-12	0.37	0.10
7	3	-11	0.20	0.11
7	3	-10	0.35	0.13
7	3	-9	0.64	0.14
7	3	-8	0.77	0.10
7	3	-7	0.63	0.12
7	3	-6	0.56	0.07
7	3	-5	0.95	0.06
7	3	5	0.63	0.08
7	3	6	0.48	0.09
7	3	7	0.39	0.13
7	3	8	0.35	0.13
7	3	9	0.49	0.12
7	3	10	0.58	0.10
7	3	11	0.39	0.11
7	3	12	0.06	0.09
7	3	13	0.12	0.18
7	3	14	0.43	0.14
7	3	15	0.30	0.14
9	0	-15	0.75	0.11
9	0	-14	0.69	0.11
9	0	-13	0.18	0.19
9	0	-12	0.01	0.04
9	0	-11	0.13	0.12
9	0	-10	0.44	0.13
9	0	-9	0.50	0.15
9	0	-8	0.55	0.13
9	0	-7	0.67	0.10
9	0	-6	0.84	0.05
9	0	-5	0.90	0.05
9	0	-4	0.85	0.05
9	0	-3	0.68	0.06
9	0	-2	0.48	0.09
9	0	-1	0.65	0.07
9	0	0	1.07	0.06
9	0	1	1.38	0.04
9	0	2	1.40	0.04
9	0	3	1.09	0.05
9	0	4	0.66	0.06
9	0	5	0.60	0.09

Continuation of Table 1

h	k	l	F^o	$\sigma(F^o)$
9	0	6	0.92	0.04
9	0	7	1.05	0.06
9	0	8	0.92	0.10
9	0	9	0.69	0.12
9	0	10	0.43	0.13
9	0	11	0.13	0.12
9	0	12	0.03	0.07
9	0	13	0.13	0.17
9	0	14	0.38	0.15
9	0	15	0.35	0.17
